# Steroid 21-hydroxylase gene variants and late-life depression

**DOI:** 10.1186/s13104-021-05616-6

**Published:** 2021-05-25

**Authors:** Marie-Laure Ancelin, Joanna Norton, Karen Ritchie, Isabelle Chaudieu, Joanne Ryan

**Affiliations:** 1 INM, Univ Montpellier, INSERM, Montpellier, France; 2 Centre for Clinical Brain Sciences, University of Edinburgh, Edinburgh, UK; 3 Biological Neuropsychiatry and Dementia Unit, School of Public Health and Preventive Medicine, Monash University, Melbourne, VIC Australia

**Keywords:** Late-life depression, Older adults, Population-based study, Stress, Corticosteroids, Single-nucleotide polymorphisms

## Abstract

**Objectives:**

A feature of late-life depression is alterations of the stress hormone system. The *CYP21A2* gene encodes for the steroid 21-hydroxylase enzyme which is required for the biosynthesis of mineralocorticoids and glucocorticoids, two main components of the stress response in humans. Variants in the *CYP21A2* gene could influence risk of late-life depression, but this has not been examined. This study investigated possible associations between five variants in the *CYP21A2* gene and late-life depression in 1007 older community-dwelling men and women.

**Results:**

In multivariate logistic regression model, significant associations were found between three single-nucleotide polymorphisms (*rs389883*, *rs437179*, and *rs630379*) and depression in women specifically (OR ranging from 1.51 to 1.68, p-values 0.025 to 0.0045), and the two latter remained significant after correction for multiple testing. Variants of the *CYP21A2* gene appear as susceptibility factors for late-life depression in a sex-specific manner, independently of somatic and neuropsychiatric comorbidity.

**Supplementary Information:**

The online version contains supplementary material available at 10.1186/s13104-021-05616-6.

## Introduction

A feature of late-life depression (LLD) is alterations of the stress hormone system [1]. Stress hormone secretion can be influenced by a number of factors such as age, sex, comorbidity, and genetic sensitivity to environmental stress [1, 2]. Recent evidence suggests that depression can be divided into a reactive subtype more vulnerable to intrinsically stress-related environmental factors and neurodevelopmental mechanisms and an endogenous subtype with a strong biological and/or genetic basis and no apparent environmental precipitants [3, 4]. Endogenous depression may involve genes related to serotonergic system and hypothalamic–pituitary–adrenal (HPA) axis [3, 5–7].

The *CYP21A2 *(cytochrome P450, family 21, subfamily A, polypeptide 2) gene encodes for the steroid 21-hydroxylase enzyme which catalyzes the conversion of progesterone to 11-deoxycorticosterone and 17-hydroxyprogesterone to 11-deoxycortisol in the biosynthesis of mineralocorticoids (aldosterone) and glucocorticoids (cortisol), the two main components of stress response in humans [8]. Mild to severe 21-hydroxylase deficiency notably causes changes to sex and adrenal hormone activities and leads to steroid hormone imbalances [9]. It can lead to a shunt away from cortisol and aldosterone synthesis to form androstenedione, which can drive the synthesis of androgens or lead to estrone via aromatase [10].

Altered cortisol and sex steroid levels and psychiatric disorders have been reported in clinical studies of young adults with 21-hydroxylase deficiency [11, 12], especially in women [13]. In older general population, Velders et al. found weak associations between some *CYP21A2* polymorphisms and cortisol secretion [14]. For three variants, an association with a broad depression phenotype (depressive symptoms combined with major depressive disorder diagnosis) was reported from a meta-analysis of genome-wide association studies [15]. None of these studies examined women specifically, although female hormones can influence both depression [16] and the HPA axis response to stress [17], especially in older adults [2]. There is also clear evidence for female specificity in the genetic basis of both depression as well as cortisol secretion and response to stress [18, 19]. However, despite the fact that stress hormones play a clear role in depression, the possible influence of genes involved in corticosteroid biosynthesis on depression is still poorly explored [20].

In this study, we investigated the relationships between *CYP21A2* genetic variants and depression in a large cohort of older adults while taking into account multiple causes of depression, including vascular factors and neuropsychiatric comorbidity. We hypothesized that genetic variation within *CYP21A2* would contribute to the risk of LLD, independently of comorbidity, and that these relationships could be modified by sex.

## Main text

### Methods

#### Design and setting of the study

Community-dwellers aged 65 years or older were selected by random sampling from electoral roles in the Montpellier district, France [21]. Ethics approval was given by the national ethics committee (Ethical Committee of Sud Méditerranée III and University Hospital of Kremlin-Bicêtre, France) and all participants provided informed consent. Participants underwent standardized clinical assessments as well as health (socio-demographic and anthropometric characteristics, lifestyle, medical history) and psychiatric interviews.

#### Genotyping

This study was based on a sample of 1007 non-demented participants who underwent depression assessment and agreed to provide buccal samples for DNA. Five polymorphisms (*rs389883, rs437179, rs429608, rs438999,* and *rs630379*) were selected based on their potential association with cortisol secretion [14] and a recent meta-analysis of depression genome-wide association studies [15]. Genotyping was performed by LGC Genomics, UK, using the KASP SNP genotyping system [22].

#### Clinical variables

Lifetime depression and anxiety disorders were diagnosed according to DSM-IV criteria [21] using the Mini-International Neuropsychiatric Interview (MINI), a standardized psychiatric examination validated in the general population [23]. The Center for Epidemiologic Studies-Depression Scale (CES-D), validated in older general population, was used to evaluate current depressive symptomatology [24]. In older adults, LLD covers a range of mild to severe depressive symptoms which does not always correspond to the DSM criteria for major depressive disorder, despite devastating consequences [25, 26]. To adequately capture this construct, case-level LLD was defined as a MINI diagnosis of current major depressive disorder or clinical level of depressive symptomatology (CES-D score ≥ 16) [22]. Cognitive impairment was defined as having a Mini-Mental State Examination (MMSE) score < 26 [27]. MMSE and MINI were administered by psychologists and psychiatric nurses and positive cases of depression were reviewed by a panel of psychiatrists. Dementia was diagnosed by a neurologist as part of a standardized examination and validated by a panel of independent neurologists [28].

#### Statistical analysis

Associations between *CYP21A2* polymorphisms and LLD were assessed using logistic regression adjusted for age and after stratification by sex. Multivariate analyses further adjusted for cognitive impairment, body mass index, cardiovascular pathologies, past major depressive disorder, and current anxiety disorder. SAS (v9.4, SAS Institute, NC, USA) was used for the statistical analyses with a significance level of p < 0.05. Given that five SNPs were investigated, the Bonferroni corrected p-value was 0.01.

## Results

One quarter of the 1007 participants were identified as having LLD (Table 1). They were more frequently women, with a lower education level and more likely to have cognitive impairment, past major depressive disorder, current anxiety disorder and to use antidepressant than non-depressed participants (p ≤ 0.004). The *CYP21A2* genotype frequencies were not significantly different from those predicted by Hardy–Weinberg equilibrium (p > 0.21 for all SNPs) (Additional file 1 Table S1). Owing to the small number of homozygotes for the minor allele of all polymorphisms (< 4%), these homozygotes were combined with the heterozygotes for analysis.

**Table 1:** Baseline characteristics of participants according to prevalent late-life depression (LLD)^a^ (N = 1007^b^)

Characteristic	No LLD (n = 751, 74.58%)	LLD (n = 256, 25.42%)	LLD vs no LLD (OR [95%CI]^c^)	Wald test P-value
Age (years) mean (sd)	71.41 (4.39)	71.97 (4.46)	1.03 [0.99; 1.06]	0.08
Sex (female)	414 (55.13)	188 (73.44)	2.24 [1.64; 3.07]	< 0.0001
Education (≥ 12 years schooling)	231 (30.76)	44 (17.19)	0.54 [0.37; 0.78]	0.001
Body mass index (kg/m^2^)				
Normal (< 25)	427 (56.86)	142 (55.47)	1	
Overweight (25–29)	275 (36.62)	89 (34.77)	1.18 [0.86; 1.63]	0.30
Obese (≥ 30)	49 (6.52)	25 (9.77)	1.61 [0.95; 2.72]	0.08
Diabetes (fasting glycemia > 7 mmol l^−1^ or treated)	51 (6.84)	15 (5.86)	1.11 [0.60; 2.04]	0.75
Cardiovascular ischemic pathologies^d^	78 (10.39)	27 (10.55)	1.17 [0.73; 1.89]	0.51
Hypertension (> 95/160 mmHg or treated)	335 (44.61)	112 (43.75)	0.97 [0.72; 1.30]	0.82
Cognitive impairment (MMSE score < 26)	62 (8.26)	40 (15.63)	1.88 [1.22; 2.89]	0.004
History of major depression	153 (20.37)	95 (37.11)	2.05 [1.49; 2.81]	< 0.0001
Antidepressant use	15 (2.00)	28 (10.94)	5.09 [2.65; 9.78]	< 0.0001
Current anxiety disorder	70 (9.32)	63 (24.61)	2.86 [1.95; 4.20]	< 0.0001

^a^Corresponds to current major depression or a CES-D score ≥ 16

^b^Except for diabetes (N = 1002)

^c^Adjusted for age (continuous), except for age (unadjusted)

^d^A history of angina pectoris, myocardial infarction, stroke, cardiovascular surgery and arteritis

In age-adjusted regression model, *rs389883*, *rs437179*, and *rs630379* were associated with an increased risk of LLD in the whole sample and in women specifically (Table 2). Women with minor alleles of *rs389883*, *rs437179*, and *rs630379* had a 51–68% increased risk of depression compared with homozygotes for the major allele, and the two latter remained significant after Bonferroni correction. The same pattern was observed in the multivariate-adjusted regression models or when changing the depression outcome to also include participants not reaching our criteria for LLD but currently using antidepressants (Table 3).

**Table 2:** Logistic regression analysis for the association between *CYP21A2* polymorphisms and prevalent late-life depression (LLD)^a^ in the whole sample and according to sex

SNP and genotype	Whole sample (N = 1007)				Men (N = 405)				Women (N = 602)			
	No LLD	LLD	OR [95% CI]^b^	p	No LLD	LLD	OR [95% CI]^b^	p	No LLD	LLD	OR [95% CI]^b^	p
	%	%			%	%			%	%		
*rs389883, n*	*733*	*252*			*327*	*68*			*406*	*184*		
AA	64.26	58.73	–	–	61.47	63.24	–	–	66.50	57.07		
AC + CC	35.74	41.27	1.32 [0.98; 1.77]	0.07	38.53	36.76	0.98 [0.57; 1.69]	0.94	33.50	42.93	1.51 [1.05; 2.16]	0.025
*rs437179, n*	*739*	*251*			*331*	*65*			*408*	*186*		
GG	64.28	58.57	–	–	60.42	64.62	–	–	67.40	56.45		
GT + TT	35.72	41.43	1.34 [0.99; 1.80]	0.056	39.58	35.38	0.87 [0.50; 1.52]	0.63	32.60	43.55	1.61 [1.13; 2.30]	0.009
*rs429608, n*	*722*	*251*			*326*	*65*			*396*	*186*		
GG	67.73	69.32	–	–	66.87	73.85	–	–	68.43	67.74		
AG + AA	32.27	30.68	0.93 [0.68; 1.27]	0.64	33.13	26.15	0.70 [0.38; 1.28]	0.25	31.57	32.26	1.03 [0.71; 1.50]	0.86
*rs438999, n*	*746*	*255*			*335*	*68*			*411*	*187*		
TT	77.08	78.43	–	–	77.61	79.41	–	–	76.64	78.07		
CT + CC	22.92	21.57	0.92 [0.65; 1.30]	0.64	22.39	20.59	0.87 [0.45; 1.65]	0.66	23.36	21.93	0.93 [0.61; 1.41]	0.73
*rs630379, n*	*746*	*254*			*336*	*67*			*410*	*187*		
GG	65.28	59.06	–	–	61.90	67.16	–	–	68.05	56.15		
GT + TT	34.72	40.94	1.35 [1.00; 1.82]	0.047	38.10	32.84	0.82 [0.47; 1.44]	0.49	31.95	43.85	1.68 [1.17; 2.40]	0.0045

^a^Corresponds to current major depression or a CES-D score ≥ 16

^b^Model adjusted for age (and sex for whole sample)

**Table 3:** Multivariate logistic regression analysis^a^ for the association between *CYP21A2* polymorphisms and prevalent late-life depression (LLD) in women (N = 602)

SNP and genotype	No LLD^b^ %	LLD^b^ %	OR [95% CI]^b^	p^b^	No LLD^c^ %	LLD^c^ %	OR [95% CI]^c^	p^c^
*rs389883, n*	*406*	*184*			*394*	*196*		
AA	66.50	57.07	–	–	67.26	56.12		
AC + CC	33.50	42.93	1.47 [1.01; 2.13]	0.04	32.74	43.88	1.58 [1.10; 2.29]	0.014
*rs437179, n*	*408*	*186*			*396*	*198*		
GG	67.40	56.45	–	–	67.93	56.06		
GT + TT	32.60	43.55	1.59 [1.10; 2.30]	0.01	32.07	43.94	1.65 [1.14; 2.38]	0.0075
*rs429608, n*	*396*	*186*			*384*	*198*		
GG	68.43	67.74	–	–	68.23	68.18		
AG + AA	31.57	32.26	0.96 [0.65; 1.42]	0.85	31.77	31.82	0.95 [0.64; 1.39]	0.78
*rs438999, n*	*411*	*187*			*399*	*199*		
TT	76.64	78.07	–	–	76.69	77.89		
CT + CC	23.36	21.93	0.81 [0.53; 1.26]	0.36	23.31	22.11	0.84 [0.54; 1.29]	0.42
*rs630379, n*	*410*	*187*			*398*	*199*		
GG	68.05	56.15	–	–	68.84	55.28		
GT + TT	31.95	43.85	1.65 [1.14; 2.39]	0.008	31.16	44.72	1.77 [1.23; 2.56]	0.002

^a^Model adjusted for age, ischemic pathologies, cognitive impairment (MMSE < 26), body mass index, past major depression, and current anxiety disorder

^b^Corresponds to current major depression or a CES-D score ≥ 16

^c^Corresponds to current major depression or a CES-D score ≥ 16 or currently using antidepressant or treated with antidepressants (combined prevalence 33.4%)

## Discussion

Three of five SNPs examined, *rs389883, rs437179,* and *rs630379*, were associated with a more than 50% increased risk of LLD in women specifically, independently of potential physical and mental health-related confounders. Our finding of female-specific associations aligns with what has been reported in the literature, with both age and sex modifying the cortisol response to challenge. A meta-analysis reported a consistent effect of age upon cortisol responses which was almost three-fold stronger in older women than men [2]. Female-specific genetic determinants of morning cortisol levels have also been reported in a genome-wide study [19]. Sex hormones can influence the HPA-axis response to stress, and a more potent reaction to stress has been observed in females [17] and they can also influence depression. In clinical studies, specific alterations in cortisol and sex steroid levels as well as psychiatric disorders were reported in women with 21-hydroxylase deficiency [13]. In our community-dwelling population of older adults, we have previously shown that variants of the *CYP19A1* gene, which codes for aromatase, the key enzyme in the conversion of androgen to estrogen, were susceptibility factors for LLD in women specifically [29]. We also just reported that variants of the *CYP11B1* gene coding for 11-β hydroxylase, the next enzymatic step after CYP21A2 in steroidogenesis pathway, were susceptibility factors for LLD in women [30]. Hence, several pathways related to steroidogenesis involving the major classes of steroids (progestogens, mineralocorticoids, glucocorticoids, androgens and estrogens), may shape to LLD in women. This may help explain sex-different vulnerability.

Our findings align with those of a recent meta-analysis of genome-wide association studies having linked these variants to depression. Indeed, *rs389883* was one of the 8 novel genome-wide significant index SNPs for broad depression phenotype which were replicated in a large population-based cohort [15]. *Rs389883* was in high linkage disequilibrium with *rs630379* and with the non-synonymous coding SNP *rs437179* [15]. These three SNPs were investigated for their association with diurnal cortisol secretion in older general population [14], but they failed to reach significant levels (p’s 0.07–0.10). In neither study were potential sex-differences examined.

In congenital adrenal hyperplasia, mutations in the *CYP21A2* gene can cause varying degrees of 21-hydroxylase activity loss leading to a range of phenotypes (from androgen excess for the milder form to virilization or “salt-wasting” with cortisol and mineralocorticoid deficiency for the most severe) [9]. However, the exact functional consequences of the variants in our study have not been examined.

## Conclusion

Our findings provide new epidemiological support for *CYP21A2* polymorphisms as independent susceptibility factors for LLD in a sex-specific manner. However, depression is a complex trait and it is likely that in addition to the effects of single genetic variants, depression is influenced by gene-environment and gene–gene interactions. Additional studies are needed to confirm these findings in other populations and to investigate the functionality of the associated variants.

## Limitations

Limitations of our study include bias from excluding institutionalized participants and those with missing data. This may have decreased the overall power of the study. Despite the relatively large size of the sample, we could not examine specifically minor homozygotes due to their low frequencies. Our study focused on a specific candidate gene, rather than considering a number of genes involved in corticosteroid biosynthesis, or using a genome-wide association approach. Although candidate-gene studies are hypothesis driven rather than having the discovery approach of genome-wide studies, they remain of value to investigate known genes with strong a priori biological rationale. They are also more appropriate for relatively smaller studies and help to reduce the risk of false positives that was minimized by correcting for multiple comparisons.

## Supplementary Information

**Additional file 1**: **Table S1**. Number and frequency of *CYP21A2* genotypes.

## Data Availability

The datasets analysed during the current study are available from the corresponding author on reasonable request.

## Abbreviations

CES-D: Center for Epidemiologic Studies-Depression Scale
*CYP21A2*: Cytochrome P450, family 21, subfamily A, polypeptide 2
HPA: Hypothalamic–pituitary–adrenal
LLD: Of late-life depression
