# Supplementary material for: Steroid 21-hydroxylase gene variants and late-life depression
Source: BMC Res Notes. 2021 May 25;14:203. doi: 10.1186/s13104-021-05616-6 (PMC8147346; doi:10.1186/s13104-021-05616-6)
Supplement: Supplementary file 1 — Additional file 1: Table S1. Number and frequency of CYP21A2 genotypes. [file 13104_2021_5616_MOESM1_ESM.docx]

**Additional file 1**

**Table S1** Number and frequency of *CYP21A2* genotypes

| **SNP and genotype** | **n** | **%** | **p^a^** |
| --- | --- | --- | --- |
| *rs389883* | *985* |  |  |
| AA | 619 | 62.84 | 0.409 |
| AC | 329 | 33.40 |  |
| CC | 37 | 3.76 |  |
| *rs437179* | *990* |  |  |
| GG | 622 | 62.83 | 0.307 |
| GT | 332 | 33.54 |  |
| TT | 36 | 3.64 |  |
| *rs429608* | *973* |  |  |
| GG | 663 | 68.14 | 0.215 |
| AG | 287 | 29.50 |  |
| AA | 23 | 2.36 |  |
| *rs438999* | *1001* |  |  |
| TT | 775 | 77.42 | 0.683 |
| CT | 210 | 20.98 |  |
| CC | 16 | 1.60 |  |
| *rs630379* | *1000* |  |  |
| GG | 637 | 63.7 | 0.283 |
| GT | 329 | 32.90 |  |
| TT | 34 | 3.40 |  |

^a^Hardy-Weinberg equilibrium p-value
